# Neurophysiological identification and differentiation between the motor and sensory roots in pediatric spinal cord lipoma surgery

**DOI:** 10.1007/s00381-024-06673-5

**Published:** 2024-12-06

**Authors:** Katharina Lutz, Timothy Müller, Sebastian Grunt, Cordula Scherer, Martin U. Schuhmann, Mazen Zeino, Sonja Vulcu, Arsany Hakim, Jonathan Wermelinger, Pablo Abel Alvarez Abut, Katarzyna Pospieszny, Andreas Raabe, Philippe Schucht, Kathleen Seidel

**Affiliations:** 1 Department of Neurosurgery, Inselspital, Bern University Hospital, and University of Bern, Bern, Switzerland; 2 Division of Neuropediatrics, Development and Rehabilitation, Department of Pediatrics, Inselspital, Bern University Hospital, and University of Bern, Bern, Switzerland; 3 Department of Pediatric Surgery, Inselspital, Bern University Hospital, and University of Bern, Bern, Switzerland; 4 Section of Pediatric Neurosurgery, Department of Neurosurgery, University Hospital of Tübingen, Tübingen, Germany; 5 Division of Pediatric Urology, Department of Pediatric Surgery, Inselspital, Bern University Hospital, and University of Bern, Bern, Switzerland; 6 University Institute of Diagnostic and Interventional Neuroradiology, Inselspital, Bern University Hospital, and University of Bern, Bern, Switzerland

**Keywords:** Spinal cord lipoma, Intraoperative neurophysiology, Nerve root stimulation, Mapping, Reflex

## Abstract

**Background:**

Radical resection of spinal cord lipomas reduces the rate of re-tethering. Current conventional neurophysiological mapping techniques are not able to differentiate between crucial motor nerve roots and sensory roots. Enhanced differentiation could contribute to complete resection. We present our experience with a double-train paradigm to differentiate between motor and sensory roots.

**Methods:**

In children undergoing spinal cord lipoma resection, the double-train mapping paradigm was used with an inter-train interval of 60 ms. Given the longer recovery time due to the H-reflex, a single muscle response was presumed to be elicited from a sensory root, and a double muscle response from a motor root. The primary endpoint was postoperative neurological outcome and bladder function at discharge.

**Results:**

We included 8 children undergoing 10 lipoma resections between 2016 and 2023. Double-train mapping was used in all cases. Motor and sensory roots were clearly differentiated in 6 cases and altered the course of surgery in 4 cases.

Post-surgery, no sensory and motor function worsened within 3 months. Bladder function was stable in six and improved in two children. In two patients, bladder function worsened slightly at 3 months and 6 months, at which point one patient was re-operated on for re-tethering.

**Conclusion:**

Intraoperative mapping with the double-train paradigm reliably differentiated between motor and sensory nerve roots. Informing the surgeon on the specific function of a tethering root may help to maximize resection without risking major neurological deficits.

## Introduction

According to the classification of Pang et al., the type of spinal lipoma in children (dorsal, transitional, terminal, or chaotic) affects the complexity of the surgery [1–3].

Complete neurosurgical resection or maximal reduction of spinal lipomas in children has significant risks of postoperative neurological deficits. Due to anatomical alterations caused by the lipoma, neuronal structures like the conus, motor, and sensory nerve roots are difficult to discern from fibrous adhesions within the lipoma and from the lipoma tissue itself [1, 4–10].

Intraoperative neurophysiological monitoring (IOM) and mapping help to discern neuronal tissue from adhesions and non-functional roots [11–13].

Mapping techniques of nerve roots via a hand held probe are used to identify critical neural structures within the surgical field and provide surgeons with valuable insights into complex neuroanatomy [6, 11–20]. To improve the temporal coverage of stimulation, mapping might be applied continuously via an electrified surgical instrument such as a CUSA [21]. However, the interpretation of sensory rootlet stimulations remains problematic even in experienced centers [12].

In lipoma surgery, the identification of nerve roots and the corresponding surgical approaches present different levels of complexity. On a first level, any structure, which upon stimulation does not elicit any neurophysiological response, can be sacrificed if required for detethering. On a second level, functional considerations are important for intra-operative decision-making. While the conus as well as the motor roots and all roots involved in bladder and sphincter control must be preserved at all costs, lumbar sensory roots may be sacrificed if required for detethering, as the ensuing sensory deficits typically do not lead to significant loss of quality of life. Hence, a lumbar sensory nerve root may be sacrificed if necessary to obtain detethering.

For peripheral nerve stimulation, Hoffmann et al. described in 1918 that electrical stimulation of the posterior tibial nerve in the popliteal fossa evoked long-latency responses of the triceps surae in humans [22]. After this discovery, these reflex responses were termed the H-reflex [23, 24]. In 1943, Lloyd reported the elicitation of “dorsal root–ventral root reflex discharges” in a cat model through a two-neuron reflex arc [25]. Since then, the technique has been applied in humans. Non-invasive transdermal electrical or magnetic stimulation as well as epidural stimulation of posterior lumbar cord structures can elicit dorsal root–ventral root reflex discharges and consequently be recorded from the muscle to which the motoneuron discharge is directed as a monosynaptic reflex [26–28]. This has been called posterior root–muscle reflexes (PRM reflexes) [26–28]. Minassian et al. refined the technique to differentiate between sensor-motor PRM and motor M responses [29]. Applying a double pulse protocol, they could find that the PRM was depressed at an inter-train interval (ITI) of 50 ms. This refractory period excluded the possibility that the responses were produced by direct activation of alpha-motoneurons in the ventral horn or anterior roots [30, 31].

In a previous study, we applied the same principle of double stimulation to distinguish responses after stimulation of the dorsal column (DC) from the corticospinal tract (CST) during surgery of intramedullary spinal cord tumors [32]. However, to our knowledge, until now, no one has applied the technique to distinguish motor from sensory nerve roots in spinal lipoma or tethered cord surgery. In this proof of concept study, we report about our preliminary experience in applying this protocol to spinal cord lipoma surgery in children as an additional tool to guide the resection strategy.

## Methods

### Study design and data collection

This study included children (aged 0–18 years) who underwent spinal cord lipoma resection in our institution, Inselspital Bern, between January 2016 and February 2023, in whom differentiation between the motor and sensory root was considered to be beneficial for surgical guidance.

Patients with very small terminal lipomas (including fatty filum) not in contact with the spinal cord were excluded from the study, as, in such cases, resections were performed without detailed mapping and did not require distinction between the motor and sensory nerve roots. We retrospectively evaluated our pre- and postoperative magnetic resonance imaging (MRI) data, together with clinical and IOM records.

MRIs were evaluated by a board-certified neuroradiologist, measuring both lesion diameter and volume in pre- and postoperative images using sagittal precontrast T1 with a slice thickness of 2–3 mm. Diameter was measured utilizing the multi-planar reconstruction tool within our institution’s picture archiving and communication system. Volume was calculated by manually delineating the intraspinal lesion using the level tracing and paint tools in 3D Slicer version 4.11. Pre- and postoperative lipoma volumes were measured in milliliters (mL) and used to categorize the extent of resection as gross total (GTR), near total (NTR), or subtotal resection (STR). Furthermore, lipomas were classified according to Pang et al. [16].

Clinical data were retrospectively extracted from patient records. We assessed demographic parameters (including age at the time of surgery and sex), as well as preoperative status and postoperative clinical outcomes (including neurological and orthopedic status and bladder function) immediately following surgery, at 3 months, and beyond 6 months post-surgery. A urodynamic or video-urodynamic study was conducted preoperatively as well as at 3 and 6 months post-surgery. Additionally, we documented all complications and recorded recurrences.

Approval was granted by the regional ethics committee (BASEC-Nr: 2023–00352).

### Surgical procedure and intraoperative neurophysiological monitoring

Lipoma resection followed our established microsurgical procedures and was carried out with the assistance of neurophysiological guidance. All patients underwent the surgical procedure under total intravenous anesthesia with IOM and mapping. Our surgical aim was a total safe lipoma resection or, if this was not achievable, maximum safe lipoma reduction. All patients received an expansion duroplasty with an allogenic graft.

**Fig. 1:**
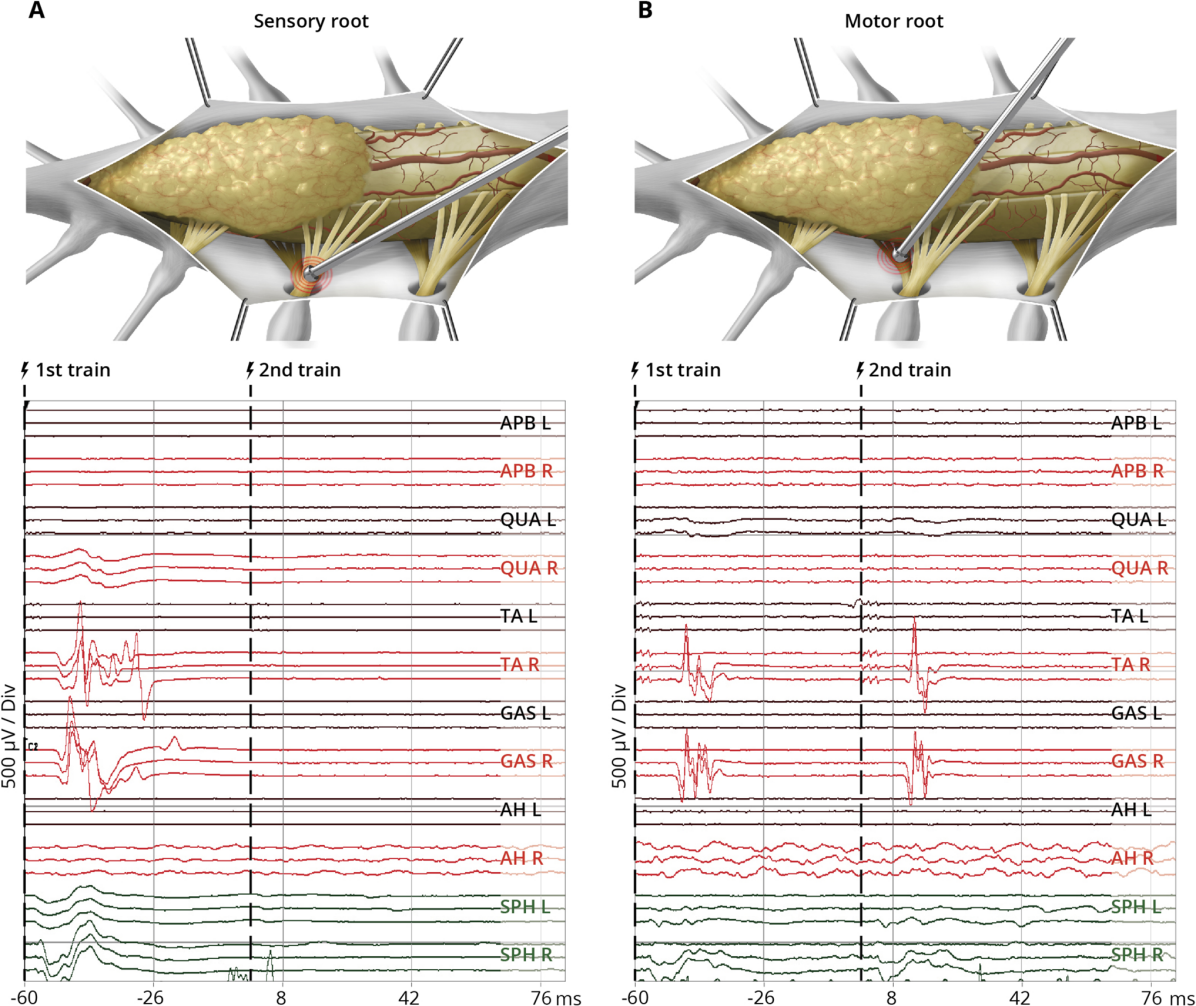
Schematic illustration of the intraoperative findings of a probe used for nerve root stimulation using the double-train mapping technique during lipoma surgery and the corresponding IOM recordings (based on real representative intraoperative triggered EMG recordings). Upper row: Schematic illustration of the operative field during surgery of a spinal lipoma with intraoperative mapping using a bipolar concentric probe to identify the sensory root (**A**) and motor root (**B**). Lower row: Intraoperative responses after double-train stimulation (  ) of nerve roots with elicitation of ipsilateral muscle responses for tibialis anterior and gastrocnemius muscle with a single response pattern indicating the sensory root (left panel) and a double response pattern indicating the motor root (right panel). L, left; R, right; APB, M. abductor pollicis brevis; QUA, M. quadriceps femoris; TA, M. tibialis anterior; GAS, M. gastrocnemius; AH, M. abductor halluces; SPH, M. sphincter ani

IOM was performed with SEPs, MEPs, pudendal SEPs, and BCR. For mapping, we used a bipolar concentric probe (Inomed 522101, Inomed Co, Emmendingen, Germany) with a stimulation intensity ranging from 2 to 0.1 mA with a pulse duration of 0.5 ms, interstimulus interval of 4.0 ms, and a train of 3–5 pulses. For intraoperative distinction between motor and sensory roots, we used a newly developed double-train mapping paradigm with an ITI of 60 ms (Fig. 1) [32, 33]. Given the longer recovery time due to the H-reflex, a single muscle response was presumed to be elicited from a sensory root (Fig. 1A), and a double muscle response from a motor root (Fig. 1B).

Further, we defined the spinal level according to the observed response pattern based on the classification from Schirmer et al. (2011: Table 5) [34] and anatomical correlation. We are aware that traditional myotomal maps may not fully represent the individual variations seen in clinical practice. Schirmer et al. [34] demonstrated this variability in muscle activation for each spinal nerve root, highlighting the overlap between adjacent roots and a multiple innervation of certain muscles. Their data revealed a non-linear distribution of nerve stimulation outcomes across different patients, emphasizing that traditional textbook definitions of myotomal innervation often fail to capture the complex and individualized patterns observed during surgery.

### Outcome

The primary endpoint of our study was the clinical outcome (motor, sensory, urological, and orthopedic) immediately after surgery, categorized as improved, stable, or worsened. These terms were used comparatively, indicating changes in clinical status from previous assessments. At the 3-month mark, outcomes were compared to preoperative clinical status, while comparisons at > 6 months were made against the 3-month clinical status. Secondary endpoints included IOM results (stable, decline, loss of signal), extent of resection, intraoperative change of the surgical strategy, recurrence, and clinical outcome (motor, sensory, urological, and orthopedic) at 3 months and beyond 6 months postoperatively.

We also analyzed the influence on recurrence and complications.

### Statistical analyses

We employed an ordinal scale to assess neurological, urological, and orthopedic data both before and after surgery. The extent of resection was defined as GTR (80–100%), NTR (50–80%), or STR (< 50%). The IOM data were categorized as (I) stable, (II) deterioration, or (III) loss. Neurological, urological, and orthopedic outcomes were categorized as evident deficits or normal status preoperatively and whether they were worse postoperatively compared to the preoperative status. The sample size was too small to enable statistical tests that could effectively detect potential benefits.

## Results

### Patient population and characteristics

We employed the double-train stimulation technique in 10 interventions for spinal cord untethering and resection of spinal lipomas in 8 pediatric patients; 2 cases were redo surgeries. Median age at the time of surgery was 19 months (range 7–160 months) (Table 1). In all primary cases, the presence of cutaneous indicators (such as tail-like skin appendages) raised suspicion of spinal lipomas, which were confirmed by a comprehensive whole spine MRI.

**Table 1:**
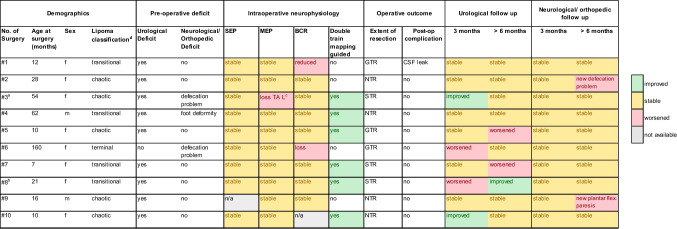
Pre- and postoperative clinical and surgical data for all performed surgeries

^a^Re-op of surgery #2

^b^Re-op of surgery #7

^c^Loss of tibialis anterior muscle, left side

^d^According to Pang et al. [16]

*GTR* gross total resection, *NTR* near total resection, *STR* subtotal resection, *CSF* cerebrospinal fluid, *MEP* motor evoked potential, *SEP* sensory evoked potential, *BCR* bulbocavernosus reflex

In accordance with the classification system introduced by Pang et al. [1], our cohort comprised 4 transitional, one terminal, and 5 chaotic-type lipomas (Table 1).

The follow-up period after surgery ranged from 6 months to 7 years (median, 46 months).

### Intraoperative findings

IOM was implemented in all 10 surgical interventions. SEPs were found to be inapplicable from the beginning in one case, whereas they exhibited sustained stability in all other cases. MEPs were consistently recorded as stable (*n* = 9), apart from one significant change observed in surgical case #3, where the MEP of the tibialis anterior was lost on the left side (Table 1).

During one of the surgeries, the BCR was undetectable. The BCR remained stable in 7 cases, and loss or significant reduction of the BCR was observed during the remaining 2 interventions (Table 1). In the cases of BCR loss, there was a decrease in bladder function directly postoperatively.

**Fig. 2:**
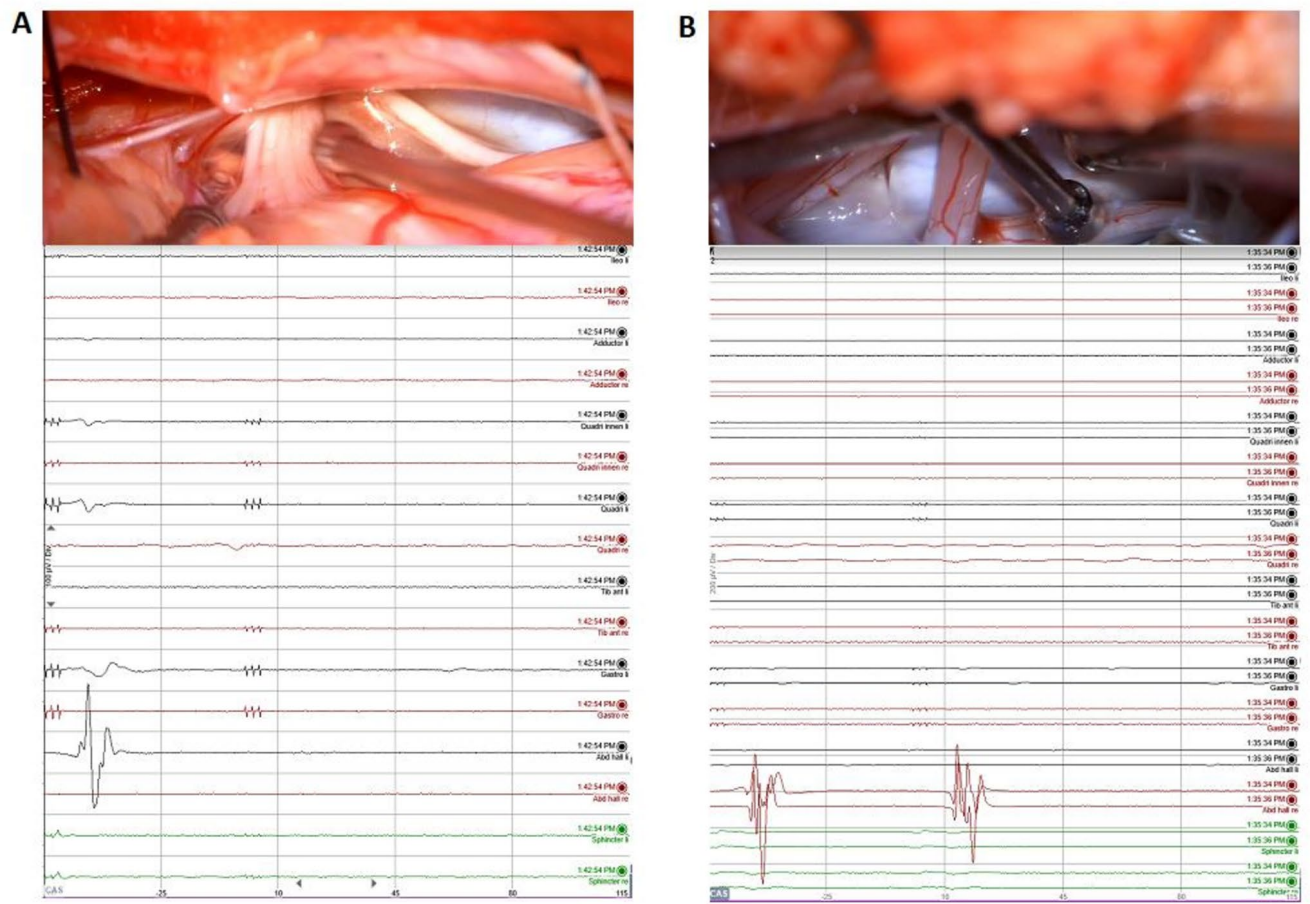
Intraoperative stimulation (upper row) and the corresponding intraoperative triggered EMG recording (lower row). Left column (**A**): After stimulation with the double-train paradigm, a single response with the highest amplitude in the abductor halluces (AH) on the left side is recorded, indicating the sensory (posterior) S1 root. Right column (**B**): After stimulation with the double-train paradigm, a double response in the AH muscle on the right side can be recorded, indicating the motor (anterior) S1 root

**Fig. 3:**
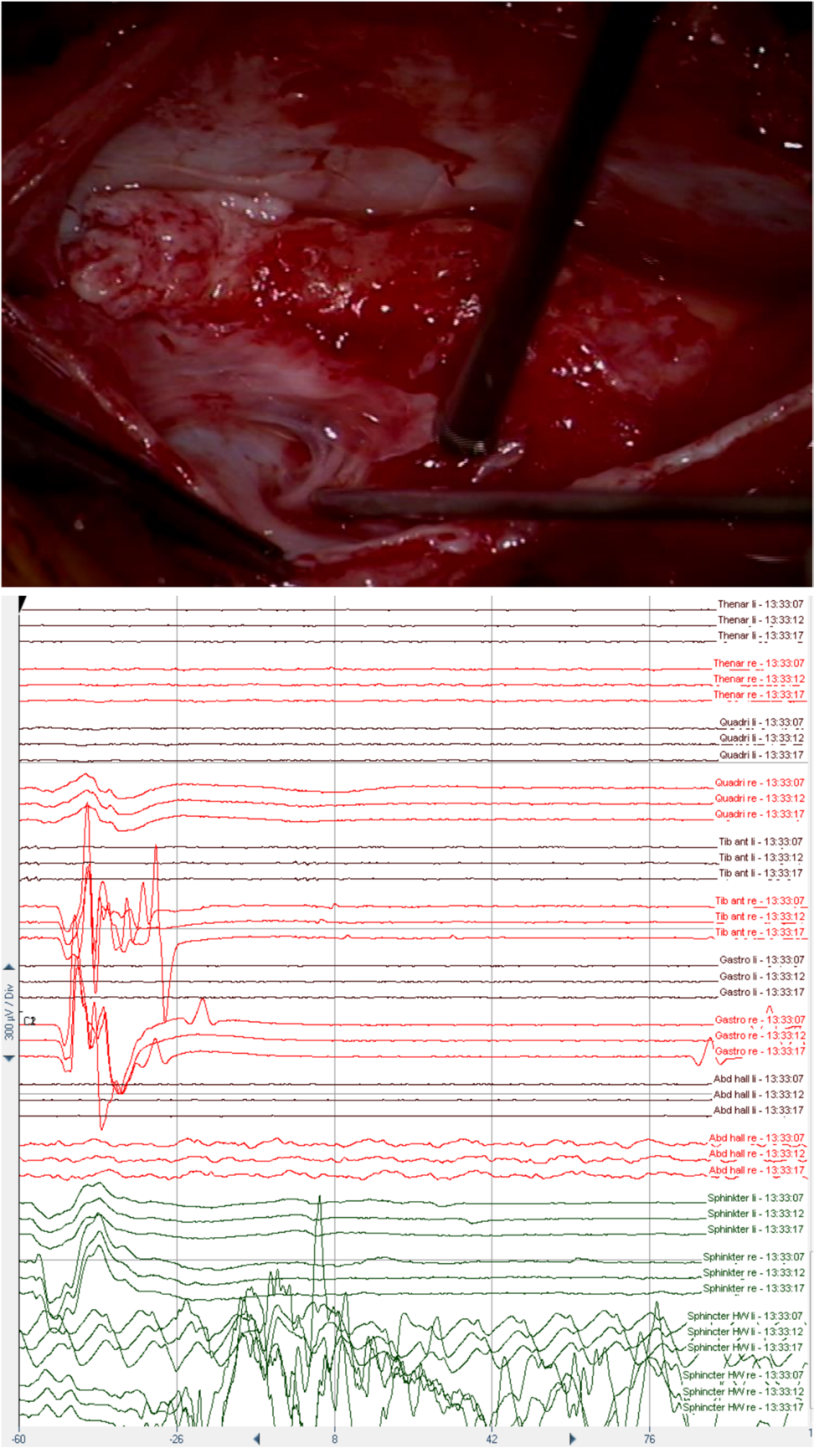
Intraoperative stimulation (upper row) and the corresponding intraoperative triggered EMG recording (lower row). After stimulation with the double-train paradigm, a single response can be recorded in the tibialis (TA) and gastrocnemius (GAS) muscles on the right side spreading to other sensory levels. The highest amplitude response in TA and Gastro on the right side corresponds to the posterior L5 root (according to Schirmer et al. [34]). Please note also the single response in the needles of the sphincter (upper two sets of 3 green lines) and late responses in the hook wire recordings of the sphincter (lower green lines). Those responses may be related to sensory afferents to the sphincter muscle

**Fig. 4:**
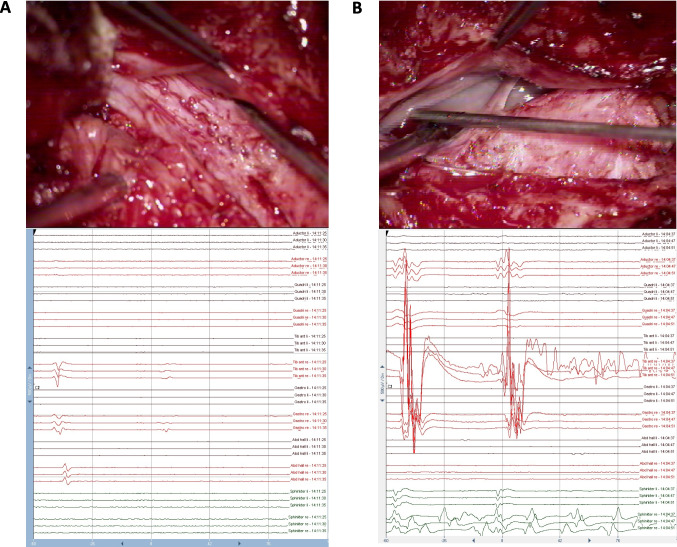
Intraoperative stimulation (upper row) and the corresponding intraoperative triggered EMG recording (lower row). Left column (**A**): After stimulation with the double-train paradigm, a single response in the TA, GAS, and abductor halluces muscles on the right side is recorded, indicating the sensory (posterior) S1 root (according to Schirmer et al. [34]). A small second response can be observed in the TA (and GAS), probably due to suprathreshold stimulation (4 mA) [27, 29]. Right column (**B**): After stimulation with the double-train paradigm, a double response in the adductor femoris, quadriceps, tibialis anterior (TA), and gastrocnemius (GAS) muscles can be recorded, with the highest amplitude response in the TA muscle on the right side indicating the motor (anterior) L5 root (according to Schirmer et al. [34])

Results from stimulation directly influenced the surgical strategy, advancement, and extent of untethering. The double-train mapping technique was applied in all cases and was used extensively in 6 of them (Table 1). In those 6 cases, the motor and sensory roots were clearly identified as being sensory or motor using the double-train method. The method significantly guided and influenced the surgical strategy in 4 of these 6 surgeries. It involved cutting of clear small anatomical roots leaving the lipoma tissue at critical areas of tethering, if there was no response in repetitive stimulation or a single response (thus indicating sensory roots) above the S1 level. For intraoperative recordings with the double-train method, see Figs. 2, 3, and 4.

Identification of sensory roots that could be sacrificed clearly influenced the surgical approach in one case. However, we spared all large roots running within the fat that had a single or double response. Finally, tissue with an unclear anatomical structure with no response was resected.

### Clinical outcome

#### Sensory and motor outcome

The immediate postoperative sensory and motor outcome was excellent (Table 1). In the case with intraoperative loss of MEP to the anterior tibial muscle, there was no postoperative weakness of this muscle (Table 1).

During the long-term follow-up (> 6 months), one patient developed M4 paresis of the left plantar flexion muscles (Table 1), without sensory deficits. An updated MRI of the spine demonstrated stable findings. The patient was treated conservatively using an ankle–foot orthosis.

#### Urological and anal sphincter outcome

Preoperative urodynamic assessments revealed neurogenic bladder dysfunction in 7 of the 8 patients. Throughout the early postoperative period, 6/8 patients (8/10 surgeries) maintained a stable bladder function at the 3-month follow-up (see Table 1), including the one with a reduction of BCR during surgery.

This patient with intraoperative loss of BCR, who was the only one with normal findings in the preoperative urodynamic study, showed a correlating postoperative deterioration of bladder function (neurogenic detrusor overactivity).

For more details, see Table 1.

#### Orthopedic outcome

Prior to surgery, one patient presented with a progressive neurogenic foot deformity (cavovarus foot). The postoperative evaluation showed that this condition remained stable. No new orthopedic impairments were observed in any other patient (Table 1).

### Complications

No intraoperative complications were noted. During the immediate postoperative period, one patient developed a cerebrospinal fluid leak, which was successfully treated with a compressive dressing and secondary suture (Table 1).

### Extent of resection

GTR, NTR, and STR were achieved in 3, 4, and 3 resections, respectively. There was no obvious correlation of GTR or NTR to deficits in the immediate postoperative period, or of STR to recurrence; however, the numbers are much too small for detecting trends.

### Recurrence

In 2 children, a reoperation was performed, one 25 and one 13 months after the initial surgery. In both cases, a secondary bladder function impairment occurred, one late, at almost 2 years, and one at 11 months after the initial surgery, and repeat imaging revealed a significant re-tethering of the residual lipoma.

## Discussion

Our series comprised 8 children undergoing 10 surgical interventions for spinal lipoma under neurophysiological monitoring and mapping with an additional introduced method of double-train mapping for identification of nerve roots. The clear identification of motor and sensory roots was consistently documented in 6 cases using the double-train method. Identification of sensory roots that could be sacrificed clearly influenced the surgical approach in one case.

As well known, GTR and NTR are associated with better long-term outcome regarding recurrent tethered cord syndrome [35, 36]. However, there is disagreement in the literature regarding the indications and timing of surgery, particularly for prophylactic surgery [1, 4–9, 14]. The safety of the child should be the highest priority. The concept behind enhancing the identification of nerve root characteristics is that it might increase the extent of resection while preserving crucial neurological functions. Unfortunately, we believe that the “threshold-technique” will not be effective for distinguishing between sensory and motor nerve roots, as the excitation thresholds for motor and sensory roots in individual cases are not known, and the pathology may affect the roots differently.

### Anatomical guidance in lipoma surgery

The ongoing debate regarding the indications for surgery for spinal lipomas highlights the need to develop and enhance methods that will improve the safety profile of the procedure by adding more knowledge about the altered anatomy. The aim is to enable a maximum safe lipoma reduction or even complete resection depending on the type of lipoma. The identification of anatomical landmarks is of utmost importance, especially in the context of preservation of function while simultaneously achieving maximal detethering. Understanding which anatomical structures perform which function not only increases patient safety, but also leads to better long-term clinical outcomes. In lipoma surgery, various anatomical landmarks can guide the surgeon during the different stages of resection as expertly demonstrated by Pang et al. [16].

The surgical approach begins with addressing the extraspinal and extradural lipoma, tracing the lipoma through the muscle fascia to the dura. After the borders of the extradural lipoma are exposed (partially through the removal of laminae), the dura is opened. Following this, the intact dorsal medulla above the lipoma is revealed. The dural edges are then exposed, and the lipoma is resected along the so-called white plane. Depending on the type of lipoma, the surgery can be anatomically very challenging due to rotation and other factors, putting exiting nerve roots especially at risk [16]. The atypical course of the roots indicates that a profound understanding of anatomy and careful surgical execution are sometimes insufficient to differentiate between sensory and motor roots. Known mapping techniques are utilized during the surgery to identify nerve roots, either sensory or motor, but a clear differentiation is not always possible (Fig. 2).

### Neurophysiological guidance in lipoma surgery

As lipomas alter the anatomy, it is not safe to rely solely on anatomical landmarks to discern structures, especially if nerve roots are traveling within the lipoma. In addition, aberrant and/or non-functional nerve roots are frequently encountered in complex or chaotic lipomas. For this reason, many centers employ IOM during spinal cord lipoma surgery [13, 16, 18]. The minimum requirement is direct motor (root) stimulation, which may be regarded as standard of care. Additionally, tibial SEPs, MEP of key leg and foot muscles (depending on the nerve roots involved), and anal sphincter MEPs are advisable. Further IOM options are BCR monitoring and pudendal SEPs to decrease the risk of bladder function impairment.

Stimulation of long tracts can be applied to distinguish between the lipoma and the spinal cord (e.g., when finding the beginning of the “white plane”) [16]. While dissecting the dural edges and the “crotch,” it is essential to ensure that the nerve roots are not accidentally cut. Here, the use of neurophysiological mapping for nerve root identification is recommended [16, 19].

**Fig. 5:**
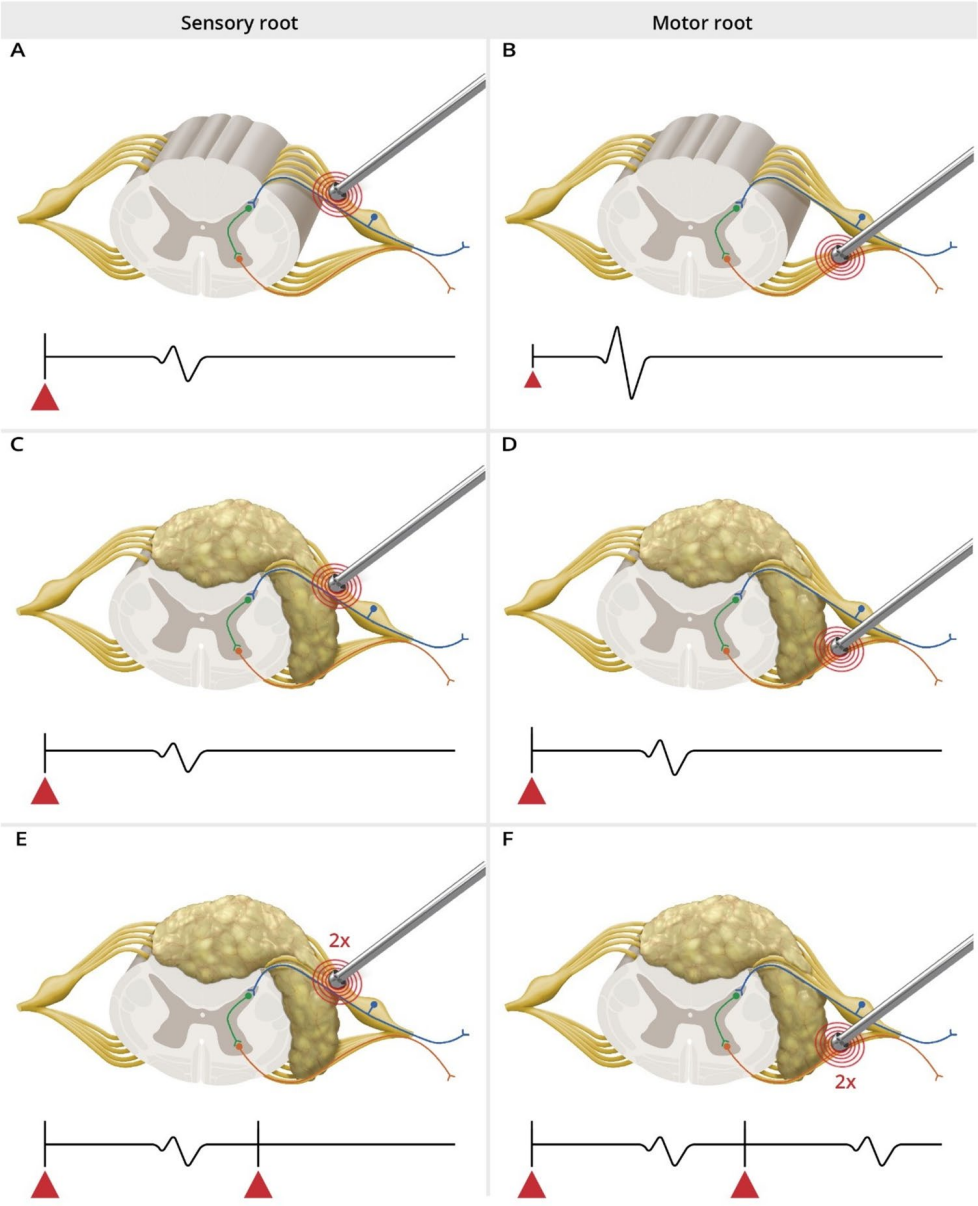
Schematic illustration of IOM responses to the threshold- and the double-train stimulation method in sensory (dorsal) and motor (ventral) nerve roots (blue color sensory neuron, green color interneuron, and orange color motor neuron). Upper row (**A** and **B**): “threshold method” in unaffected roots: (**A**) indicates a longer latency and lower amplitude with higher current (▲) stimulation compared to motor root stimulation (**B**). Middle row: “threshold method” in the presence of a lipoma: (**C**) and (**D**) demonstrate a requirement for higher stimulation intensity (▲), resulting in longer latency and reduced amplitude in comparison to stimulation of an unaffected motor root. The response from motor root stimulation (**D**) might be nearly identical to that for a sensory root stimulation (**A**) in an unaffected root. Lower row: “double-train method” in the presence of a lipoma: The first response in (**E**) and (**F**) is similar. After the second pulse train, only the motor root (**F**) shows a second EMG response. No second EMG response is elicited after stimulation of the sensory root, due to the longer recovery time (details in the text). ▲ = current intensity of the stimulus (bigger triangle = higher intensity)

Various techniques for the identification of motor nerve roots have already been used and described. Stimulation of nerve roots can be performed with high precision using a concentric bipolar stimulation probe, due to its specific electrical field. Stimulation currents of 0.3 to 3.0 mA with a pulse duration of 0.2–0.5 ms and a repetition rate of 0.5–2 Hz are used [12, 16]. Intraoperative identification of a ventral (motor) or dorsal (sensory) root may be achieved neurophysiologically using the “threshold method” [10, 19] (Fig. 5). This method involves inducing a compound muscle action potential (CMAP) through stimulation of the ventral (motor) nerve root with a low current intensity (classically: 0.3–1.5 mA, Fig. 5, right column). Stimulation of the dorsal (sensory) nerve root requires a higher current intensity that will then trigger an H-reflex (posterior root muscle reflex (PRMR)). However, this motor response has a longer latency and a lower amplitude compared to the CMAP (Fig. 5, left column) [14, 29].

This distinction between CMAP (motor root) and PRMR (sensory root) might be clear, in case the anatomy is unaffected (see Fig. 5A and B). However, altered anatomy due to a lipoma might stretch the nerve roots and can thus lead to changes in their excitability. In such cases, notably higher current intensity might be needed to elicit a CMAP from an affected motor root (Fig. 5D). The resultant CMAP might have a longer latency and a lower amplitude. Thus, it might mimic the response characteristics of a non-affected sensory root (Fig. 5A). Consequently, the surgeon might mistakenly identify this root as sensory, when in fact it is a lipoma-affected motor root.

Our study’s underlying “double-train technique” employs paired-pulse stimulation with an interstimulus interval of 60 ms. The theory behind this is based on the recovery time of the reflex arc between the sensory and motor neuron reflex arc. Stimulation of both sensory and motor roots will generate a response after the first stimulus (see Fig. 5E and F). However, stimulation of the sensory root will not generate a second response after 60 ms, because the reflex arc [37] is still in the refractory period (see Fig. 5E). In contrast, the motor root will already allow the generation of a second response (Fig. 5F). Thus, a double response is considered to be generated by a motor root and a single response pattern by a sensory root.

### The potential benefit of the “double-train technique” in lipoma surgery

Subtotal resection in lipoma surgery is a significant risk factor for re-tethering [16].

Sensory nerve roots for the lower limb might be sacrificed to reduce the lipoma bulk since the associated functional loss is minimal. Thus, in our view, clear identification of the motor and sensory roots might allow for a more extensive resection of the dorsal lipoma components. This would reduce the likelihood of postoperative re-adhesion without increasing motor deficits. Additionally, with fewer adhesions and a more extensive resection, remodeling at the end of the surgery is more manageable. This increases the arachnoid-covered surface, and a larger dural expansion graft can be employed. Both these factors are likely to reduce the risk of recurrent tethered cord syndrome [16]. The greater the intraoperative certainty through better identification of crucial functional structures, the more likely it is that these goals can be achieved.

Thus, our method represents an additional tool that could potentially contribute to improving spinal lipoma surgeries in the future by enabling more extensive resection and reducing the risk of re-tethering. This feasibility and proof of principle study in a small set of patients is a first step towards validation.

However, it is evident, that the clinical outcome does not depend solely on the double-train technique. This principle applies to various clinical factors, including motor function, sensory function, and sphincter function, among others. The outcome of the surgery is influenced by a multitude of aspects, including various surgical techniques and other IOM modalities.

## Limitations

Analysis of neurophysiological recordings is not always clear-cut. Training and experience in neurophysiology are essential to analyze the neurophysiological responses. In case of high stimulation intensity (suprathreshold stimulation), a minor second response might be observed (Fig. 4). This has been already reported by Minassian et al. during PRM studies [27, 29]. In case the stimulus intensity was increased, responses of low amplitude were elicited by the second pair of stimuli. They claimed that the refractory period of PRM reflexes depends on the stimulus intensity. Another reason might be that in case of high current intensity stimulation current spread via the CSF to neighboring anterior roots, which will be then activated as well with a lower amplitude response. Further, due to the same reasons, responses may spread to other segmental levels (Fig. 3). Additionally, Schirmer et al. [34] have already shown the variability in muscle activation for each spinal nerve root based on muscle nerve distribution observed through intraoperative nerve root stimulation. This reflects the overlap between adjacent nerve roots and the multiple innervations that certain muscles receive [34]. However, we hypothesize that it is possible to differentiate between different lower lumbar and sacral roots based on the highest amplitude responses in the corresponding muscles.

The present study has several limitations, including the small cohort size and short follow-up period. Furthermore, given the retrospective design of this study, the impact of the method remains uncertain. Due to this retrospective design depending on data from surgical reports and IOM protocols, it was unfortunately not possible to describe all surgical steps in detail. However, we aim for a prospective study to focus on the true relevance of the method compared to already existing classical IOM approaches.

Our double stimulation paradigm does not replace the need for a deep understanding of both anatomy and surgical procedures. It is simply an additional tool, which can assist surgeons in making the numerous decisions required during the resection of a lipoma. We encourage surgeons to adopt our paradigm and share their experiences. Additionally, multicenter prospective studies are needed to assess the benefit of the proposed method in lipoma surgeries.

## Conclusion

Intraoperative mapping with the double-train paradigm seems to be able to differentiate reliably between motor and sensory nerve roots during pediatric lipoma surgery. This study is a first step towards the routine use of an additional mapping tool in complex surgical interventions for spinal lipomas in children. The technique has the potential to further guide the surgeon during surgical resection, increase the extent of lipoma resection, and thus decrease the rate of re-tethering.

## Data Availability

No datasets were generated or analysed during the current study.
